# Non-postoperative Central Retinal Artery and Ophthalmic Artery Occlusion With Compression Ischemia Due to Prolonged Sedation In Prone Position

**DOI:** 10.7759/cureus.33482

**Published:** 2023-01-07

**Authors:** Sohiub N Assaf, Abdallah N Assaf, Muaz N Assaf, Joshua Pickett, John M Pierce

**Affiliations:** 1 Department of Medicine, The University of Tennessee Graduate School of Medicine, Knoxville, USA

**Keywords:** neuro-ophthalmology, hyperbaric oxygen therapy, intravenous drug use (ivdu), stroke, retinal injury, prescription drugs, blind, mri images, acute vision loss, central retinal artery occlusion

## Abstract

Central retinal artery occlusion (CRAO) after a prolonged period of lying prone is a rare condition with only a handful of cases reported, generally as a postoperative complication of spinal surgery. Only a few cases can be found describing acute visual loss following intravenous drug abuse and stupor leading to continuous pressure on the orbit while asleep. No cases can be found describing acute visual loss following the ingestion of oral sedating/antipsychotic medications. Urgent identification and workup with subsequent interventions are needed to offer the highest probability of full/partial visual restoration. Our patient presented with complete vision loss after ingesting oral antipsychotic medications leading to a prolonged sedated state in which compressive ischemia led to central retinal artery occlusion. The complex timeline regarding the patient's presentation and the implications relating to offered interventions are highlighted in this case report.

## Introduction

"Saturday night retinopathy," a term coined by Jayam et al., describes a unique case of unilateral ischemic retinopathy identified in 1974. In this case, ischemic retinopathy* *was seen in the setting of stupor that led to continuous pressure on the orbit [1]. It was presumed that the underlying pathophysiology was similar to the complications of central retinal artery occlusion (CRAO) from direct pressure on the eye during prone surgical positioning [2]. Central retinal artery occlusion often presents with acute and profound loss of vision in one eye that is usually painless. It can occasionally present with transient monocular blindness, with either a stuttering or a fluctuating course [3]. CRAO almost never occurs in both eyes simultaneously [4]. Carotid artery atherosclerosis is the most common etiology for retinal artery occlusion overall but is relatively uncommon in patients under 40 years of age [5,6]. In patients under 40, the most likely etiology is cardiogenic embolism. Other vascular causes of CRAO include carotid artery dissection, fibromuscular dysplasia, radiation injury of the carotid or retinal arteries, Moyamoya disease, and Fabry disease. Nonvascular etiologies include hematological and inflammatory diseases such as leukemia/lymphoma, sickle cell disease, giant cell arteritis, and systemic lupus erythematosus. We present a young female with complete vision loss after ingesting oral antipsychotic medications. This led the female to experience a prolonged sedated state in which compressive ischemia further led to central retinal artery occlusion with possible involvement of the ophthalmic artery.

## Case presentation

The patient is a 27-year-old female who presented to the emergency department (ED) with right monocular vision loss. Three days before her presentation, she reported falling asleep on her friend's couch, with her right eye resting on a hard metal portion of the couch frame. She reported that she had taken two tablets of quetiapine of an unknown dosage, which led to a prolonged state of sedation. She stated that she had never taken this medication before and used it in this isolated circumstance due to difficulty falling asleep. According to family members, the patient spent greater than 8-12 hours in this position. When she awoke several hours later, she found the orbit of her right eye swollen, to the extent that she was unable to open her eyelids. She immediately experienced vision loss in the right eye but attributed this to the significant swelling she was experiencing and the inability to open her right eye. She also reported a tingling sensation and numbness on her right face in an ophthalmic (V1) and maxillary branch (V2) distribution. She denied any significant pain other than soreness associated with the swelling. She denied any headaches, fevers, chills, palpitations, or any contralateral vision loss. After awakening, she delayed seeking medical attention. Three days after the inciting events, the swelling subsided enough for the patient to open her eyelid and realize that she was still experiencing vision loss. She then presented herself to a small community medical center. She underwent routine workup at the medical center, which then recommended urgent referral to ophthalmology services at our university center. Her past medical history was unremarkable, and the patient denied any history of drug abuse.

On arrival, vital signs showed a temperature of 97.2°F, a heart rate of 70 beats per minute (bpm), a respiratory rate of 18 respirations per minute (rpm), a systolic blood pressure of 106 mmHg and a diastolic blood pressure of 65 mmHg, and lastly an oxygen saturation (SpO_2_) of 98% while on room air. Physical examination showed a well-nourished young female in no acute distress. She had monocular right-sided total vision loss accompanied with marked limitation of abduction, supraduction, and infraduction and a modest abduction deficit of the right eye. She did have a loss of sensation in the forehead, face, maxillary area, and upper lip. She also displayed residual swelling with mild edema and erythema of the right lower lid. There was no sign of chemosis of the conjunctiva. Fundoscopic examination showed no significant edema but did show a cherry red macula with sharp optic disc margins and a pale retina. External ocular examination showed right ptosis and proptosis approximately 2-3 mm on the right side. The right pupil was dilated and unreactive to light and accommodation. Laboratory findings showed an unremarkable complete metabolic panel, as well as complete blood count (CBC). However, the patient's inflammatory markers were slightly elevated, and urine drug screen was positive for opioids and cannabinoids (Table 1).

**Table 1:** Laboratory results CRP, C-reactive protein; BUN, blood urea nitrogen

Laboratory parameters	Value	Reference range
Sodium	137 mEq/L	136-145 mEq/L
Potassium	4.5 mEq/L	3.5-5.0 mEq/L
Chloride	101 mEq/L	95-105 mEq/L
Bicarbonate	27 mEq/L	22-28 mEq/L
BUN	13 mg/dL	7-18 mg/dL
Creatinine	0.69 mg/dL	0.6-1.2 mg/dL
Glucose	97 mg/dL	70-110 mg/dL
Calcium	9.1 mg/dL	8.4-10.2 mg/dL
Phosphorus	3.1 mg/dL	3.0-4.5 mg/dL
Magnesium	2.1 mEq/L	1.5-2.0 mEq/L
Hemoglobin	13.4 g/dL	Female: 12.0-16.0 g/dL
Hematocrit	41.60%	Female: 36%-46%
Leukocyte count	9,400 per mm^3^	4,500-11,000 per mm^3^
Platelet count	315,000 per mm^3^	150,000-400,000 per mm^3^
Neutrophil count	4,900 per mm^3^	2,500-8,000 per mm^3^
Lymphocyte count	3,100 per mm^3^	1,000-4,000 per mm^3^
Monocyte count	500 per mm^3^	100-700 per mm^3^
Eosinophil count	800 per mm^3^	50-500 per mm^3^
Basophil count	0 per mm^3^	0-500 per mm^3^
Erythrocyte sedimentation rate	36 mm/hour	Female: 0-20 mm/hour
CRP	0.6 mg/dL	0.8-1.0 mg/dL
Opioids	Positive	
Cannabinoids	Positive	

**Figure 1:**
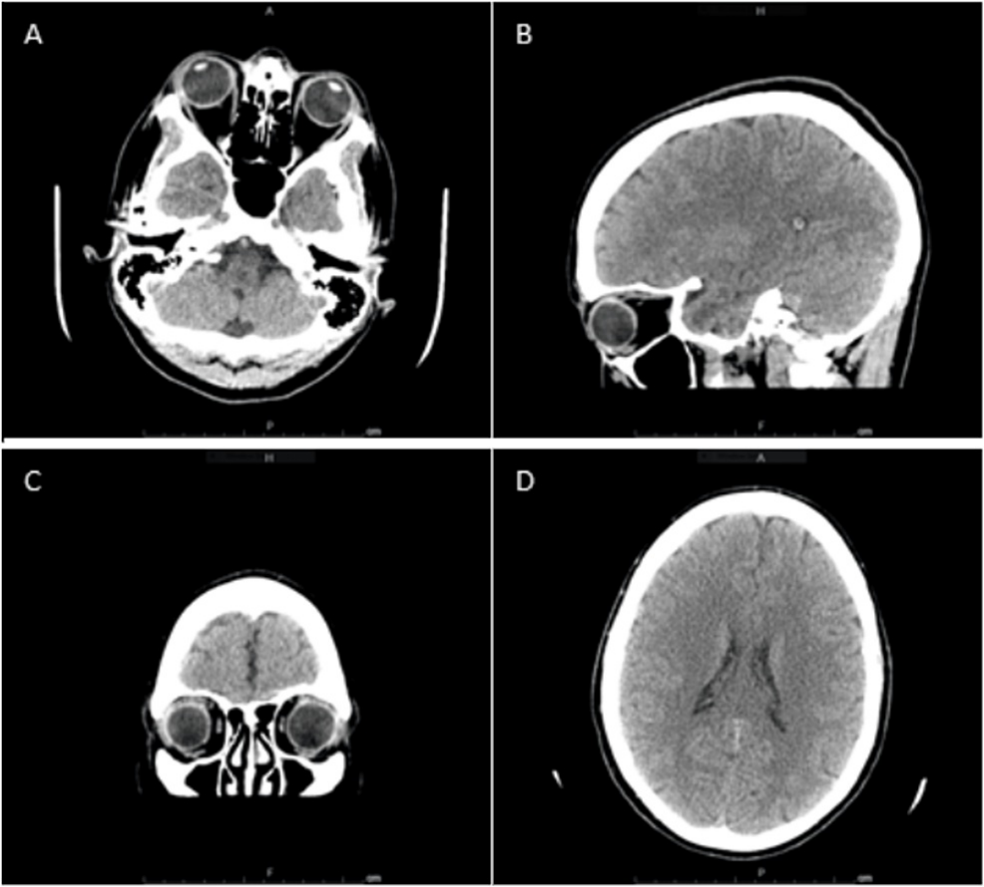
CT imaging of the head CT of the head showed no evidence of hemorrhage, no mass effect, normal attenuation characteristics of the brain parenchyma, and ventricles normal for age. (A) Transverse CT imaging showing the bilateral orbits. (B) Sagittal CT imaging showing the right orbit. (C) Coronal CT imaging showing the bilateral orbits. (D) Transverse CT imaging showing the normal ventricles CT: computed tomography

**Figure 2:**
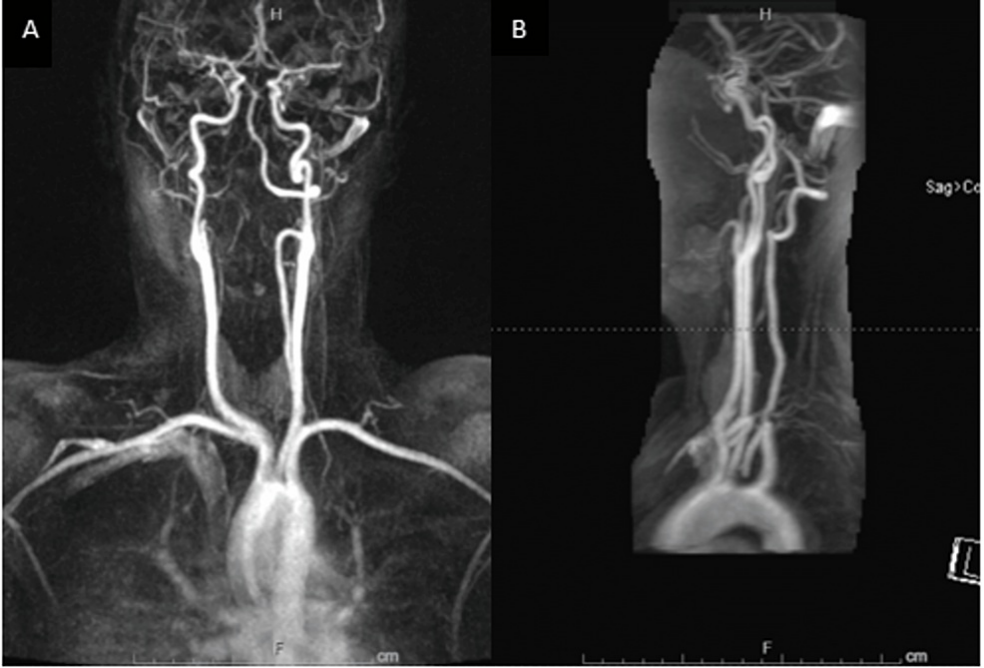
MRA imaging of the neck (A) Coronal MRA imaging showing the bilateral carotid arteries and all the major branching arteries of the neck. (B) Sagittal MRA imaging showing the carotid arteries and the vertebral arteries. Both common carotid and internal carotid arteries are widely patent. No significant atherosclerosis or stenosis at the carotid bifurcations upon NASCET criteria. The left vertebral artery is dominant. Both vertebral arteries are patent without focal stenosis. There is no significant stenosis in the cervical carotid and vertebral arteries as seen in the figure MRA, magnetic resonance angiography; NASCET, North American Symptomatic Carotid Endarterectomy Trial

**Figure 3:**
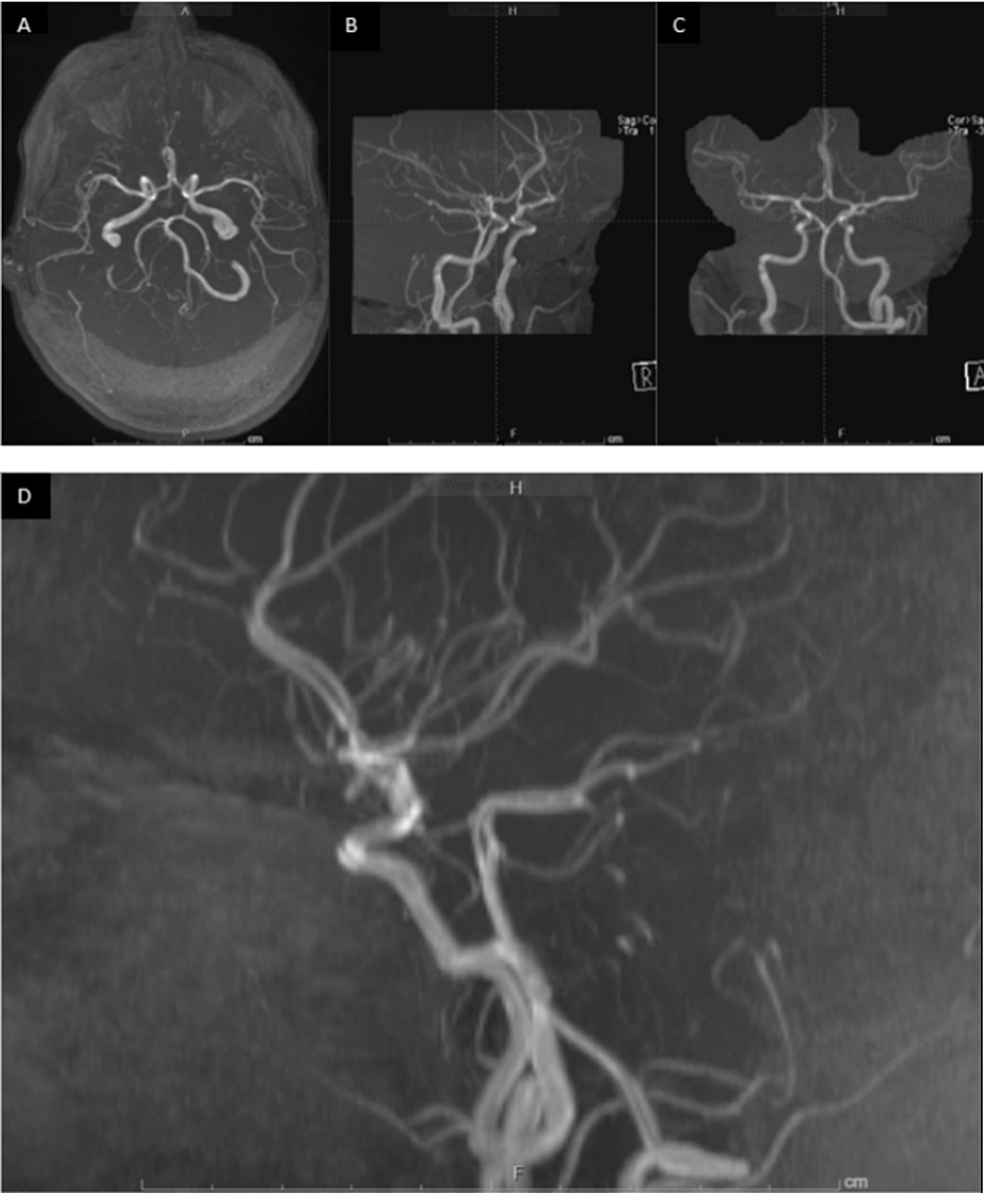
MRA imaging of the head (A) Transverse MRA imaging showing the circle of Willis. (B) Anterior-posterior MRA imaging showing the sagittal view of the circle of Willis. (C) Coronal MRA imaging showing the anterior view of the circle of Willis. (D) Detailed MRA imaging of the circle of Willis in the sagittal view, highlighting all the tributary arteries leading to the ophthalmic artery. The distal internal carotid, anterior, and middle cerebral arteries are widely patent. The distal vertebral arteries, basilar artery, and posterior cerebral arteries are widely patent. There is no evidence of aneurysm or focal stenosis. The image displays an unremarkable MRA of the head MRA: magnetic resonance angiography

**Figure 4:**
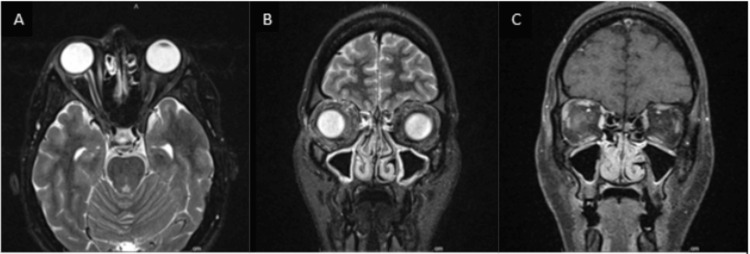
MRI imaging of the head The image shows that the right globe is intact, but there is mild right exotropia present. Subtle edema is present along the superior and lateral contour of the preseptal right orbit, which is best appreciated in the axial STIR images (A). There is a suspected mild extension of edema into the extraconal postseptal fat as in the coronal STIR image (C). Additional STIR signal abnormality with associated mild postcontrast enhancement is present along the posterior contour of the globe lateral to the optic nerve (A). There is mild asymmetric dilation of the right optic nerve sheath with flattening of the optic disc. There is suspected subtle hyperenhancement of the right optic nerve sheath as in the coronal image (B) immediately posterior to the globe. Abnormal T2 hyperintensity and hyperenhancement are present in the right superior and medial rectus muscles best seen in the coronal STIR image (C) and coronal postcontrast image (B). There is no drainable abscess identified, and no pathologic signal or pathologic enhancement is detected in the optic nerve proper. The cavernous sinuses enhance symmetrically STIR: short TI inversion recovery

Given the patient's presentation, she underwent further evaluation with computed tomography (CT) imaging, which showed no acute intracranial findings or evidence of orbital trauma (Figure 1). The magnetic resonance angiography (MRA) of the head and neck alongside the MRI of the brain was also unremarkable (Figures 2-3). The MRI of the orbits showed an unusual spectrum of signal abnormalities predominantly involving the preseptal right orbit but with the involvement of several intraorbital structures as described, including the posterior contour of the right globe, extraocular musculature, and optic nerve sheath (Figure 4).

The patient was promptly started on vancomycin and ampicillin/sulbactam for fear of atypical orbital cellulitis presentation. The patient was also treated with hyperbaric oxygen therapy; however, this was more than 24 hours after the initial presentation in the ED. The patient was transported to the ophthalmology clinic for further evaluation and was found to have a cherry red spot on the macula, suggesting this could be CRAO. It was determined that her presentation was unlikely orbital cellulitis and likely CRAO with compression ischemia of the ophthalmic artery that may have caused ischemia in the third, fourth, fifth, V1, V2, and sixth cranial nerves. The vancomycin and ampicillin/sulbactam were subsequently discontinued, and the patient was discharged on hospital day 4 with oral Augmentin to finish a seven-day course and cover any possibility of orbital cellulitis.

**Figure 5:**
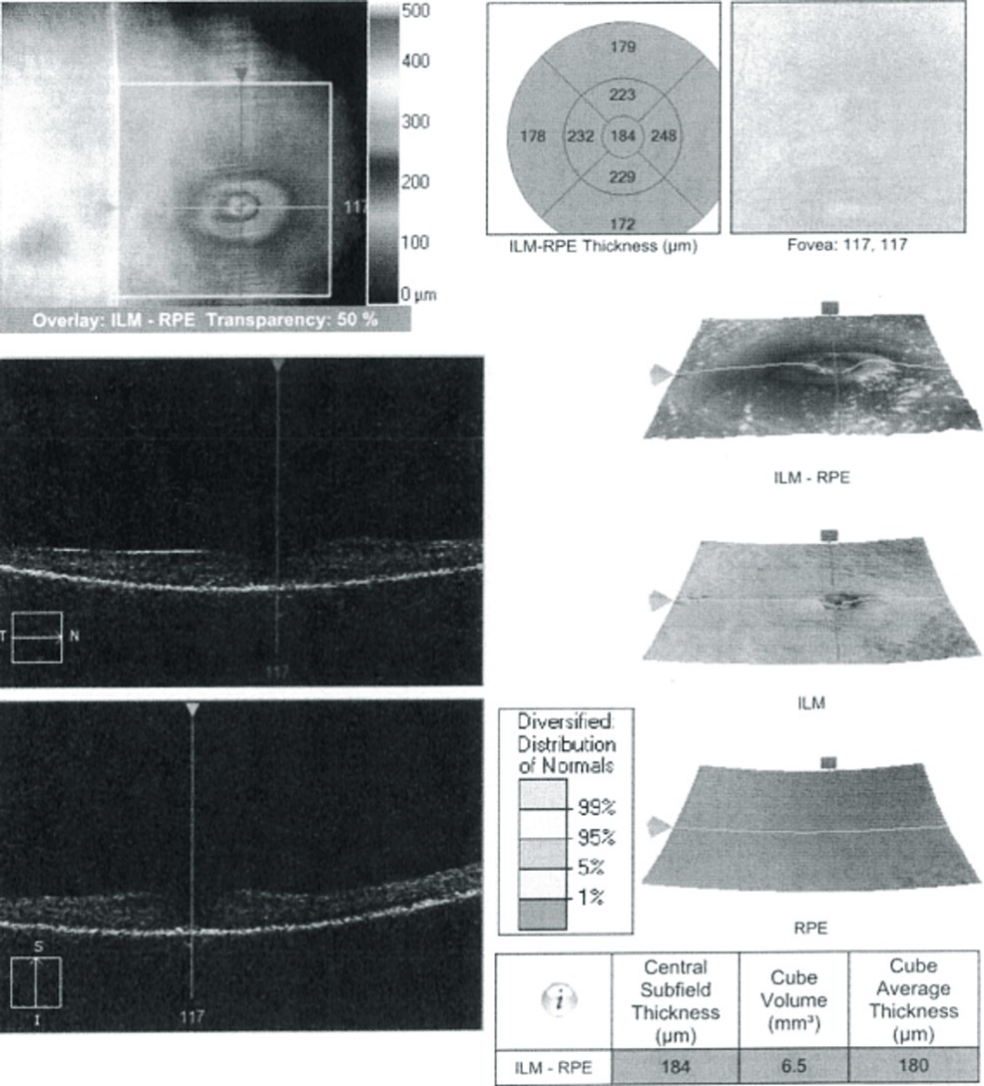
Macular thickness measured in spectral domain optical coherence tomography (SD-OCT) RPE, retinal pigment epithelium; ILM, internal limiting membrane

Patient was evaluated by neuro-ophthalmology one week after discharge and was found to have persistent vision loss in the right eye along side minimal improvement of the extraocular muscle movement. The evaluation by neuro-ophthalmology determined that the patient likely had central retinal artery occlusion secondary to the compressive ischemia sustained during her prolonged sedated state. It was also determined that the patient had either compressive involvement of the ophthalmic artery leading to ophthalmoplegia or an orbital apex-like syndrome alongside the central retinal artery occlusion. The presence of ophthalmoplegia but the absence of any etiology leading to continued increased intraorbital pressure made orbital apex syndrome less likely. During the patient's follow-up visit, it was noted that she had retinal pigment epithelial atrophy but no evidence of a macular hole or edema (Figure 5). It was noted that the right eye had generalized narrowing of the retinal vessels consistent with the diagnosis of CRAO. The patient was subsequently lost to follow-up after her final visit with the retinal specialist.

**Figure 6:**
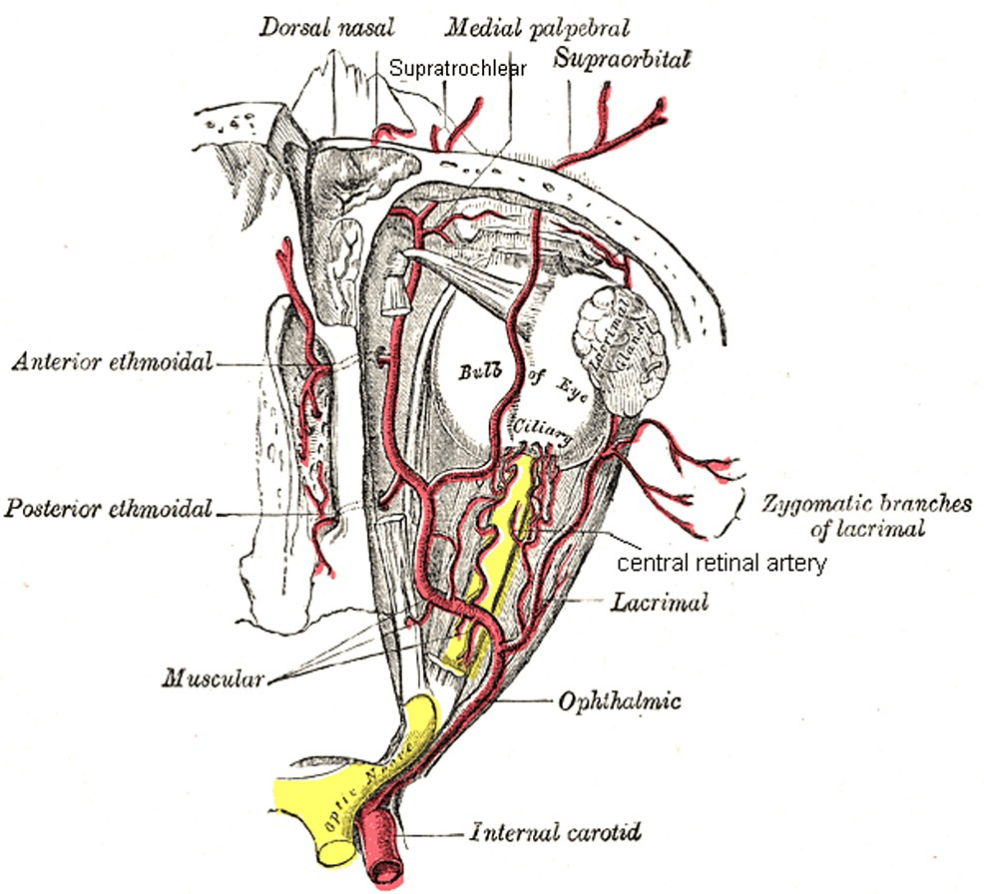
The ophthalmic artery and its branches Source: Häggström M: Medical gallery of Mikael Häggström 2014. Wiki J Med. 2014, 1:1-53. 10.15347/wjm/2014.008

The ophthalmic artery arises as the first branch from the internal carotid artery. The ophthalmic artery then divides in branches that can be categorized as ocular group and orbital group. The orbital group includes the lacrimal artery, supraorbital artery, posterior ethmoidal artery, anterior ethmoidal artery, medial palpebral artery, frontal artery, and dorsal nasal artery. The ocular group includes the long posterior ciliary arteries, short posterior ciliary arteries, anterior ciliary artery, central retinal artery, superior muscular artery, and inferior muscular artery (Figure 6).

## Discussion

Central retinal artery occlusion (CRAO) after a prolonged period of lying prone is a rare condition with only a handful of cases reported in the past. Of the cases reported, it is found to be a rare operative complication in which a patient is lying prone for the duration of an operation, inadvertently lying with pressure on the external orbit. As a postoperative complication, vision loss has an estimated incidence of 0.01%-1% depending on the type of surgery [7,8]. The incidence of CRAO due to compression ischemia is not exactly known and is most likely to be a fraction of that. This case report highlights this rare etiology of CRAO as swift identification, and subsequent intervention is necessary to increase the likelihood of vision recovery.

Our patient had a mixed picture that had presenting symptoms indicative not only of CRAO but also of ophthalmic artery occlusion. The final diagnosis favored CRAO due to the limited involvement of the other branches that feed from the ophthalmic artery and the isolated nature of her ophthalmoplegia; it was thought that this may resemble an orbital apex-like syndrome, but in the absence of any residual compressive etiology, this was determined to be less likely. What is thought to have happened is that prolonged compression led to intense intraorbital swelling and compressive ischemia to the CRAO. The subsequent swelling and compression affected the CRA and the ophthalmic artery, but then, as the swelling subsided, the residual function of the retina was lost, and the remaining function of the V1/V2 was limited alongside the innervation of the extraocular muscles. This patient's final diagnosis from ophthalmology was CRAO with ophthalmic artery involvement.

CRAO is considered an ocular emergency. Animal studies suggest that the restoration of blood flow within 90-100 minutes leads to retinal injury, while occlusions persisting for longer than 240 minutes produce massive irreversible damage to the retina [9-12]. A review of treatment options for CRAO indicated that in order to be effective, treatment should be given within six hours of ischemia onset, regardless of the method utilized [12]. Revascularization should not be delayed when indicated, but in patients with CRAO secondary to compressive ischemia, the intervention of choice would be hyperbaric oxygen therapy. Hyperbaric oxygen therapy is used to maintain the oxygenation of the retina pending reperfusion. In a small series of patients, reperfusion has shown mixed results in attempts of preserving vision [13-15].

Some late ocular complications include a neovascular response in which enough portions of the retina survive under hypoxic conditions, leading to vascular formation. Neovascularization becomes apparent after two to three months [9,16-17]. Subsequent complications include vitreous hemorrhage and neovascular glaucoma. Long-term prognosis is dependent on time to treatment and presenting baseline visual loss.

## Conclusions

Central retinal artery occlusion due to compression ischemia is an extremely rare complication of surgeries that involve prone positioning of the patient. Despite being an operative complication, it does not rule out the possibility of this occurring in a nonsurgical setting, in which a prolonged sedated state results in compressive ischemia and subsequent CRAO. Early identification and treatment are cornerstones of positive outcomes. Patients should be counseled on the judicious use of sedating medications and be made aware of this inadvertent and indirect complication of sedating medications in the outpatient setting.
